# Low dose inocula of SARS-CoV-2 Alpha variant transmits more efficiently than earlier variants in hamsters

**DOI:** 10.1038/s42003-021-02640-x

**Published:** 2021-09-20

**Authors:** Bobo Wing-Yee Mok, Honglian Liu, Shaofeng Deng, Jiayan Liu, Anna Jinxia Zhang, Siu-Ying Lau, Siwen Liu, Rachel Chun-Yee Tam, Conor J. Cremin, Timothy Ting-Leung Ng, Jake Siu-Lun Leung, Lam-Kwong Lee, Pui Wang, Kelvin Kai-Wang To, Jasper Fuk-Woo Chan, Kwok-Hung Chan, Kwok-Yung Yuen, Gilman Kit-Hang Siu, Honglin Chen

**Affiliations:** 1 Department of Microbiology and State Key Laboratory for Emerging Infectious Diseases, Li Ka Shing Faculty of Medicine, The University of Hong Kong, Hong Kong SAR, China; 2 Department of Health Technology and Informatics, Faculty of Health and Social Sciences, The Hong Kong Polytechnic University, Hong Kong SAR, China

**Keywords:** SARS-CoV-2, Viral transmission

## Abstract

Emerging variants of SARS-CoV-2 have been shown to rapidly replace original circulating strains in humans soon after they emerged. There is a lack of experimental evidence to explain how these natural occurring variants spread more efficiently than existing strains of SARS-CoV-2 in transmission. We found that the Alpha variant (B.1.1.7) increased competitive fitness over earlier parental D614G lineages in in-vitro and in-vivo systems. Using hamster transmission model, we further demonstrated that the Alpha variant is able to replicate and shed more efficiently in the nasal cavity of hamsters than other variants with low dose and short duration of exposure. The capability to initiate effective infection with low inocula may be one of the key factors leading to the rapid transmission of emerging variants of SARS-CoV-2.

## Abstract (web summary)

Bobo Wing-Yee Mok et al. observe that the Alpha (B.1.1.7) variant of SARS-CoV-2 exhibits increased competitive fitness over earlier lineages at lower doses in a hamster model of viral transmission. Their results suggest that the Alpha variant’s ability to initiate effective infection with a low dose may underlie its ability to rapidly transmit throughout a population.

## Introduction

In late 2020, a novel SARS-CoV-2 variant of concern (VOC), VOC 202012/01 (lineage B.1.1.7) was identified in the United Kingdom. This B.1.1.7 (Alpha) variant containing multiple mutations in spike^1^ has become dominant in the UK and is now rapidly spreading across multiple countries^2^. It is thought that this VOC has the potential to spread more quickly and with higher mortality than the pandemic to date^3^. Recently, using multiple behavioural and epidemiological data sources, Davies et al. estimated that the VOC 202012/01 variant (lineage B.1.1.7) has a 43–90% higher reproduction number than pre-existing variants in England^4^. In another study, Davies et al. found that among specimens collected in the UK in early 2021, higher concentrations of virus RNA were detected in nasopharyngeal swabs from B.1.1.7 infected individuals, as measured by Ct values from qRT-PCR testing^5^. However, there is a lack of experimental evidence to explain how the Alpha variant is able to spread more quickly than pre-existing variants.

The Alpha variant of SARS-CoV-2 harbours 21 nonsynonymous point mutations and three deletions in comparison to the reference genome (accession number: NC_0.45512.2)^6^. Of these, eight mutations and two deletions are in the spike protein, which interacts with the host cell receptor, angiotensin-converting enzyme 2 (ACE2), and mediates virus entry into host cells^7^. These spike mutations include the deletion ∆H69/∆V70, which has arisen in multiple independent lineages and is suggested to associate with increased infectivity and evasion of the immune response^8^; the substitution N501Y, which enhances binding affinity for the human ACE2 receptor and might affect viral transmissibility^9–11^; and the mutation P681H, which is adjacent to the S1/S2 furin cleavage site in spike and might have an impact on viral infectivity^12,13^. Interestingly, two other VOCs, P1 and B.1.351, also contain the N501Y mutation^14,15^.

The aim of this study is to provide experimental evidence to explain why the Alpha variant has increased transmissibility among the human population, compared to earlier D614G variants. Our results demonstrated that the Alpha variant exhibits increased competitive fitness over earlier D614 variants in Calu-3 cells and in hamsters. Moreover, with low dose inocula, the Alpha variant initiates more robust infectious in the upper respiratory tracts of hamsters and transmits more efficiently than earlier D614G variants.

## Results and discussion

### SARS-CoV-2 Alpha variant showed enhance replication fitness compared with earlier D614G variants

**Fig. 1:**
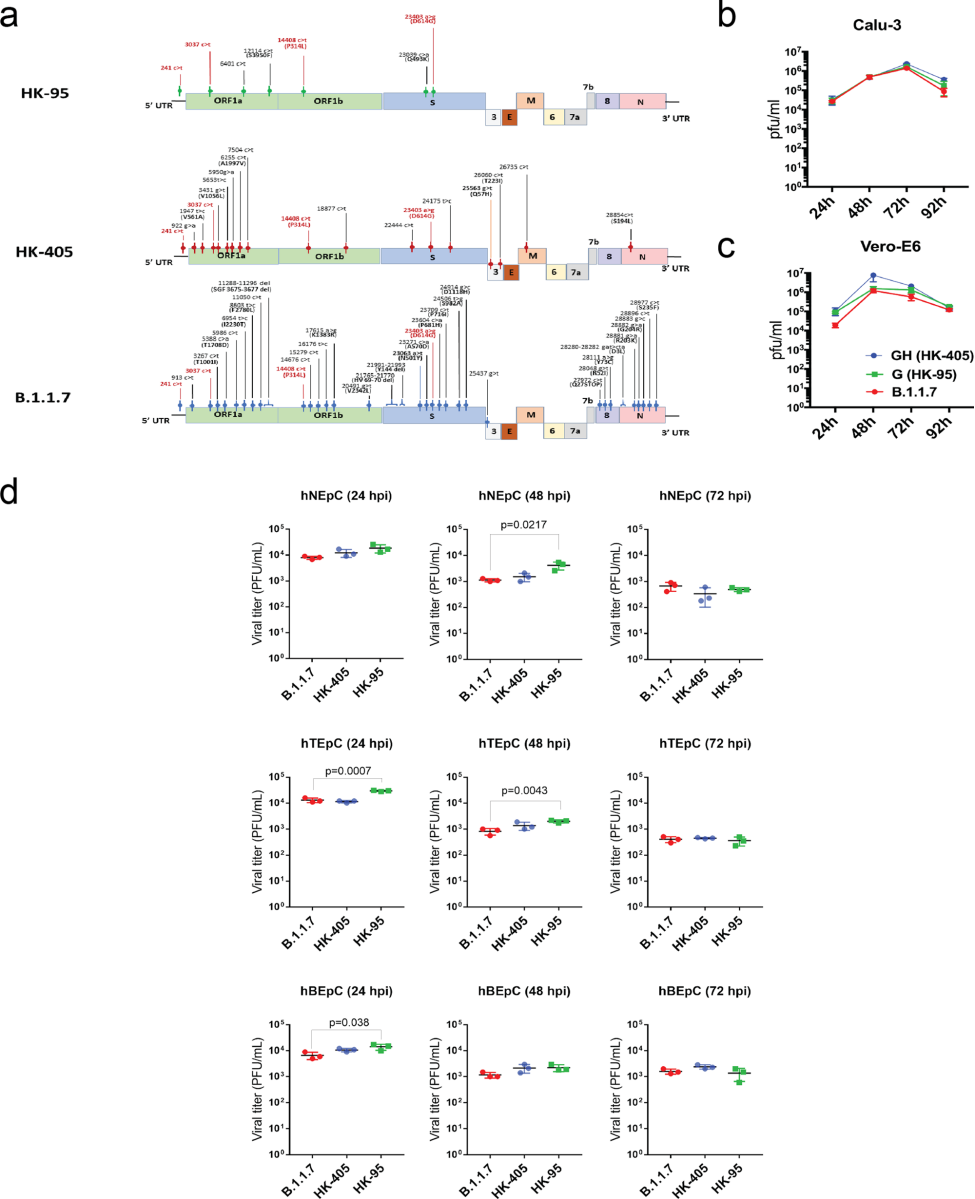
Viral growth kinetics of Sars-CoV-2 D614G variants. **a** Genomic sequence comparison of the D614G variants used in this study to the reference genome (accession number: NC_0.45512.2). Common SNPs in all thee strains are shown in red. **b** and **c** Growth curves of B.1.1.7, HK-405 and HK-95 in Calu-3 cells and Vero-E6 at a MOI = 0.01 and MOI = 0.001, respectively. Virus-infected cells were cultured at 37 °C and supernatants then harvested at the indicated time points and subjected to plaque assay in Vero E6 cells to determine the virus titre. Error bars represent mean ± SD (*n* = 3 per group for each timepoint). **d** Comparison of 24, 48 and 72 h titres between B.1.1.7, HK-405 and HK-95 infected primary nasal (hNEpC), trachel (hTEpC) and bronchial (hBEpC) epithelial cells (PromoCell) at MOI = 0.1. Triplicated titres of the viruses in the cultures from the cell lines were analysed by Student’s t-test. Horizontal lines indicate the mean ± SD of viral titre per group.

**Fig. 2:**
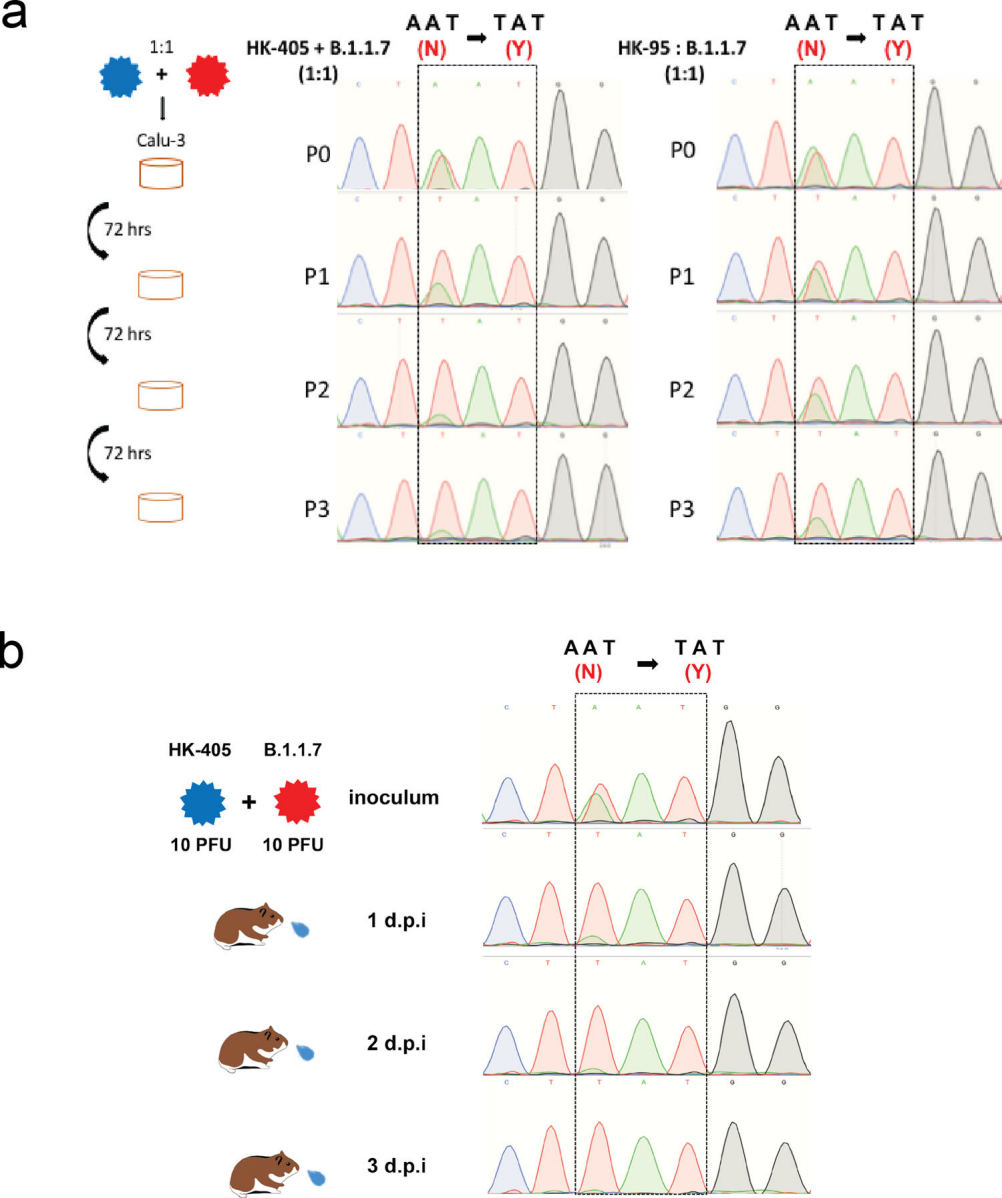
In-vitro and in-vivo competitive fitness assay. Sanger sequencing chromatograms of spike gene fragments amplified from viral samples in the competition assay. **a** Cell cultures were infected with a 1:1 mixture of two variants, as indicated, at an MOI of 0.1. The supernatants were serially passaged three times in Calu-3 cells. 901 bp fragments containing residue 501 (boxed) were amplified from the vRNA of individual samples collected from each passage (P) and sequenced. HK-95 and HK-405 contain 501N, B.1.1.7 contains 501Y. Initial inoculum was stated as P0 and P1-P3 indicated number of passages. **b** Hamsters were inoculated with a mixture of 10 PFU of each of the two viruses (HK-405 and B.1.1.7). Nasal washes were collected daily for three consecutive days and the vRNA of individual samples collected and the inoculum were sequenced.

A recent study indicated that the SARS-CoV-2 variant of concern (VOC) carrying the 501Y (B.1.1.7 or the Alpha variant) mutation showed no higher infectivity in Huh-7, Vero, and LLC-MK2 cells than ancestral D614G variants^16^. Likewise, we did not observe replication of the Alpha variant to be significantly enhanced over that of the pre-existing D614G variants (HK-95, collection date: 2020-05-15 and HK-405, collection date: 2020-12-08) (Fig. 1a) at any of the selected time-points in Vero-E6, Calu-3 cells and human primary airway cell lines (Fig. 1b–d). However, we did observe that the Alpha variant dominates in competitive fitness assays. These comparisons of replication fitness between the Alpha variant and earlier circulating strains were performed in Calu-3 cells and in hamsters through simultaneous co-infection at a 1:1 ratio with the Alpha variant and early variants of the D614G lineage (HK-405 or HK-95). After three rounds of consecutive passage at 72-h intervals in cells or 1 day postinfection in hamsters, the Alpha variant became the dominant population (Fig. 2), suggesting that those new substitutions in the Alpha variant enhance SARS-CoV-2 replication fitness in in vitro and in vivo systems.

### SARS-CoV-2 Alpha variant replicated more efficiently in the nasal cavity of hamsters than earlier D614G lineage with enhanced transmission in hamsters

**Fig. 3:**
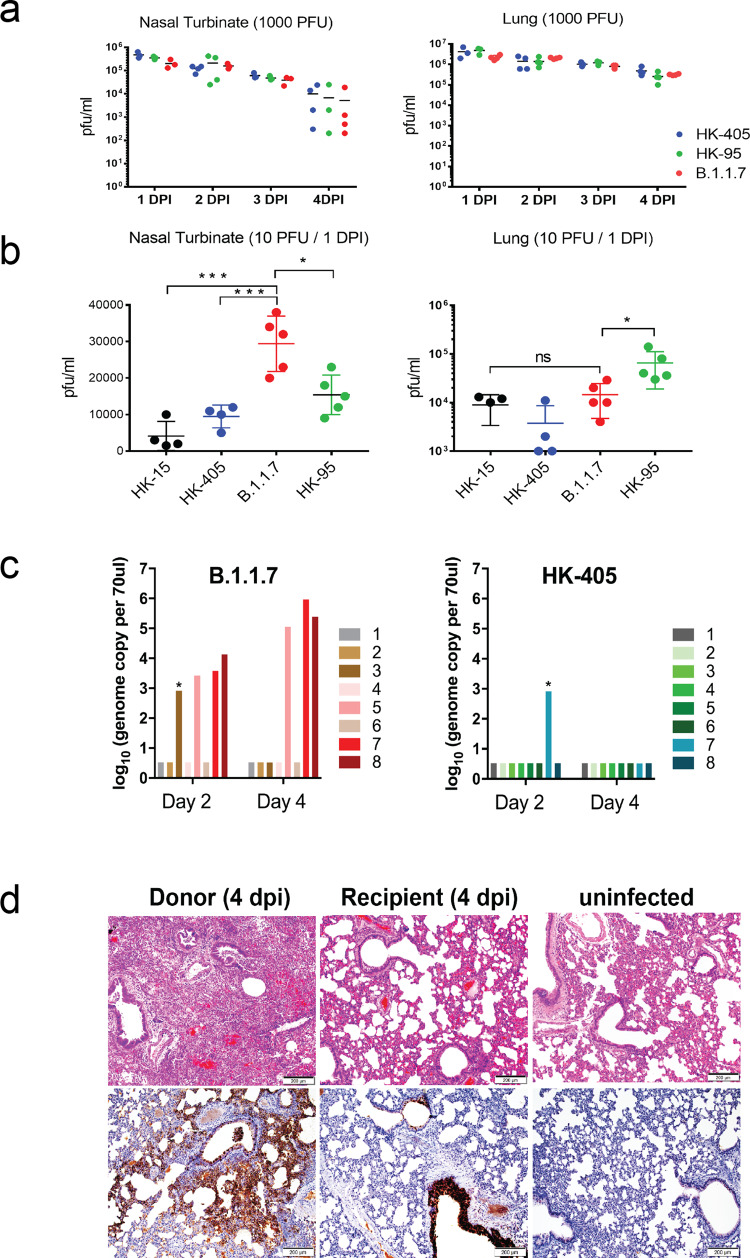
In-vivo infection studies. **a** and **b** Replication efficiency of different SARS-CoV-2 variants in nasal turbinates and lungs of hamsters. Hamsters were infected with different SARS-CoV-2 variants, as indicated. Viral titres in nasal turbinates and lungs were determined by plaque assay (PFU/ml). **a** Hamsters (14 per variant virus group) were each inoculated intranasally with 50 ul of virus stock containing 1000 PFU of virus. Three to four hamsters from each group were euthanized on each of the four consecutive days following infection for viral titration. **b** Hamsters (4-5 per group) were each inoculated intranasally with 50 ul of virus stock containing 10 PFU of virus. One non-D614G lineage variant (HK-15 (MT835141)) and three D614G lineage variants (HK-405, B.1.1.7 and HK-95) were used. Hamsters were euthanized on one day post-infection for viral titration. Horizontal lines indicate the mean (±SD) of viral titre per group. Statistical significance was calculated by Student’s t-test; * denotes *p* *<* 0.05, *** denotes *p* *<* *0.0005* and ns denotes *non-significant*. **c** Viral titres in nasal washes collected from exposed hamsters on day 2 and day 4 post-exposure. The number of positive hamsters in both exposure groups on day 4 postexposure (HK-405 vs. B.1.1.7 = 0 of 8 vs. 3 of 8), * CT value ≥35. **d** Representative images of H&E (upper) and immunohistochemistry (lower) stained hamster lung tissues taken on day 4 post-infection. The right panels were uninfected hamster lung showing normal structure and negative of viral antigen. H&E images in the left panel was B.1.1.7 infected lung (10 PFU; Donor) showing lung consolidation with massive alveoli infiltration and localised haemorrhage (open arrows). The bronchiolar lumens filled with cell debris (solid arrows) and thickened alveolar wall. Images of immunohistochemistry-stained SARS-Cov-2 nucleoprotein (brown) in donor lung tissue showed extensive viral antigen expression in the bronchiolar epithelium (open arrows) and alveoli (solid arrows). While the lung of recipient hamster (middle) only showed viral antigen in the bronchiolar epithelium (open arrows). Scale bars = 200 µM.

Next, we set up a Syrian hamster infection study to evaluate if the Alpha variant exhibits higher infectivity in vivo. 6-8-week-old male Syrian hamsters were intranasally infected with different variants of 1000 PFU per inoculum, as indicated in Fig. 3a. Infectious viral titres in upper (nasal) and lower (pulmonary) tissues were measured on four consecutive days after infection. All viruses tested replicated to similar titres in nasal turbinate and lung tissues of infected hamsters. This result is consistent with two recent studies which also found no significant alteration in infectious viral titres in samples collected from nasal washes, throat swabs and lungs from hamsters infected with different SARS-CoV-2 variants^16,17^.

**Fig. 4:**
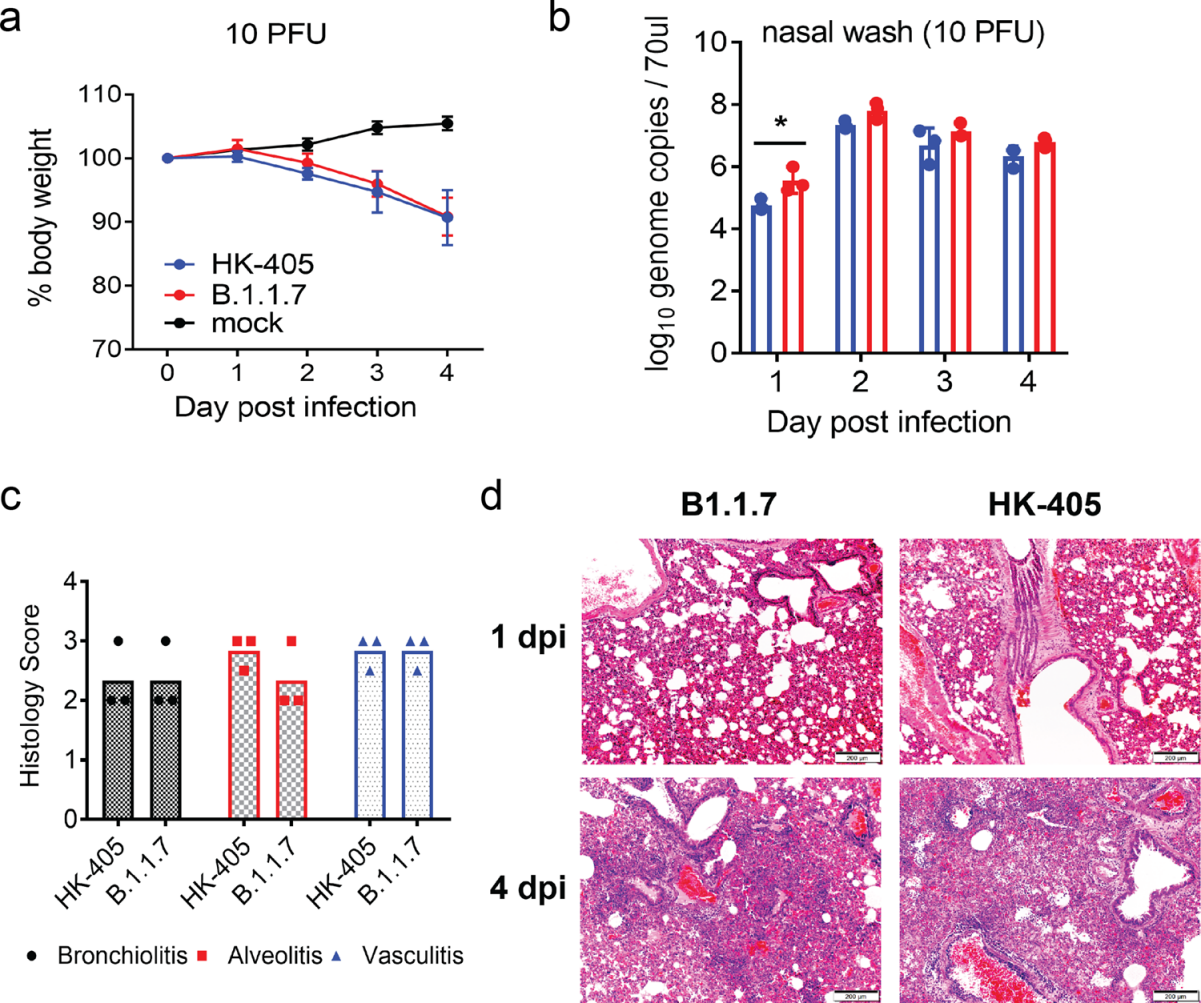
Characterisation of hamsters infected with low dose inocula of B.1.1.7 and HK-405. **a** Body weight loss of hamsters infected with low dose inocula (10 PFU) of B.1.1.7 and HK-405. **b** Viral genome copies in nasal washes of the hamsters infected with B.1.1.7 and HK-405 collected on day 1 to 4 postinfection. Individual data points and mean ± SD are shown. **c** Histological score for lungs of infected hamsters on 4 dpi. **d** Representative images of H&E-stained hamster lung (left superior lobes) taken on 1 and 4 dpi. On 1 dpi, both viruses infected lung showed alveolar congestion. But on 4 dpi, diffuse alveolar infiltration, exudation and localized haemorrhage were observed after both viruses infection. Bronchiolar luminal cell debris (solid arrows) and vascular infiltration (open arrows) were also observed. Scale bars = 200 µM.

Given that hamsters are highly susceptible to SARS-CoV-2 infection^18^, intranasal infection with high-titre inocula may hamper discrimination of differences in the infectivity and replication efficiency of variants^19^. In fact, by titrating the infection dosage (10-fold dilution) of the inocula administered to hamsters, we observed that viral replication in nasal tissues of infected hamsters had already plateaued with infection doses of 100 PFU and upwards, even on day one post-infection (Supplementary Figure 1). Humans are exposed to varying doses of infectious particles during SARS-CoV-2 transmission and some exposures may fail to establish effective infection due to insufficient infectious particles. We reasoned that SARS-CoV-2 variants, which can initiate effective infection with fewer infectious particles are likely to transmit more efficiently than other variants requiring more infectious particles. To test this, we performed hamster infection and transmission studies using only 10 PFU per inoculum (as illustrated in Supplementary Figure 2). Interestingly, infectious viral loads in nasal turbinates of hamsters were found to be significantly higher on day one post-infection with the Alpha variant compared to the other strains, whereas similar viral loads were observed in lungs of all infected hamsters, except for those inoculated with HK-95, which exhibits higher viral titres in lungs, although with large variations between replicates (Fig. 3b). Accordingly, the Alpha variant also showed enhanced transmissibility to naive hamsters compared with HK-405 (Fig. 3c). Surprisingly, we observed a striking histological difference between the Alpha variant infected lung (10 PFU; Donor) and the Alpha variant recipient lung at day 4 postinfection, with more severe pathological changes observed in the donor hamsters. Also, immunohistochemistry staining showed extensive viral antigen in donor lung tissue in the bronchiolar epithelium and alveoli, while the lung of recipient hamster showed viral antigen only in the bronchiolar epithelium (Fig. 3d). This result demonstrated that the route of exposure (direct inoculation vs indirect contact transmission) and initial dosage of the viral inoculum can largely affect the response of animals to infection. However, no apparent difference of body weight loss (Fig. 4a), viral gRNA copies in nasal wash (from 2 dpi onwards) (Fig. 4b) and histopathology (Fig. 4c & d) was observed between HK-405 and the Alpha variants, indicating that the pathogenicity of the Alpha variants is comparable to the pre-existing D614G variants.

### Calu3 responses to SARS-CoV-2 Alpha variant drive less macrophage activation

**Fig. 5:**
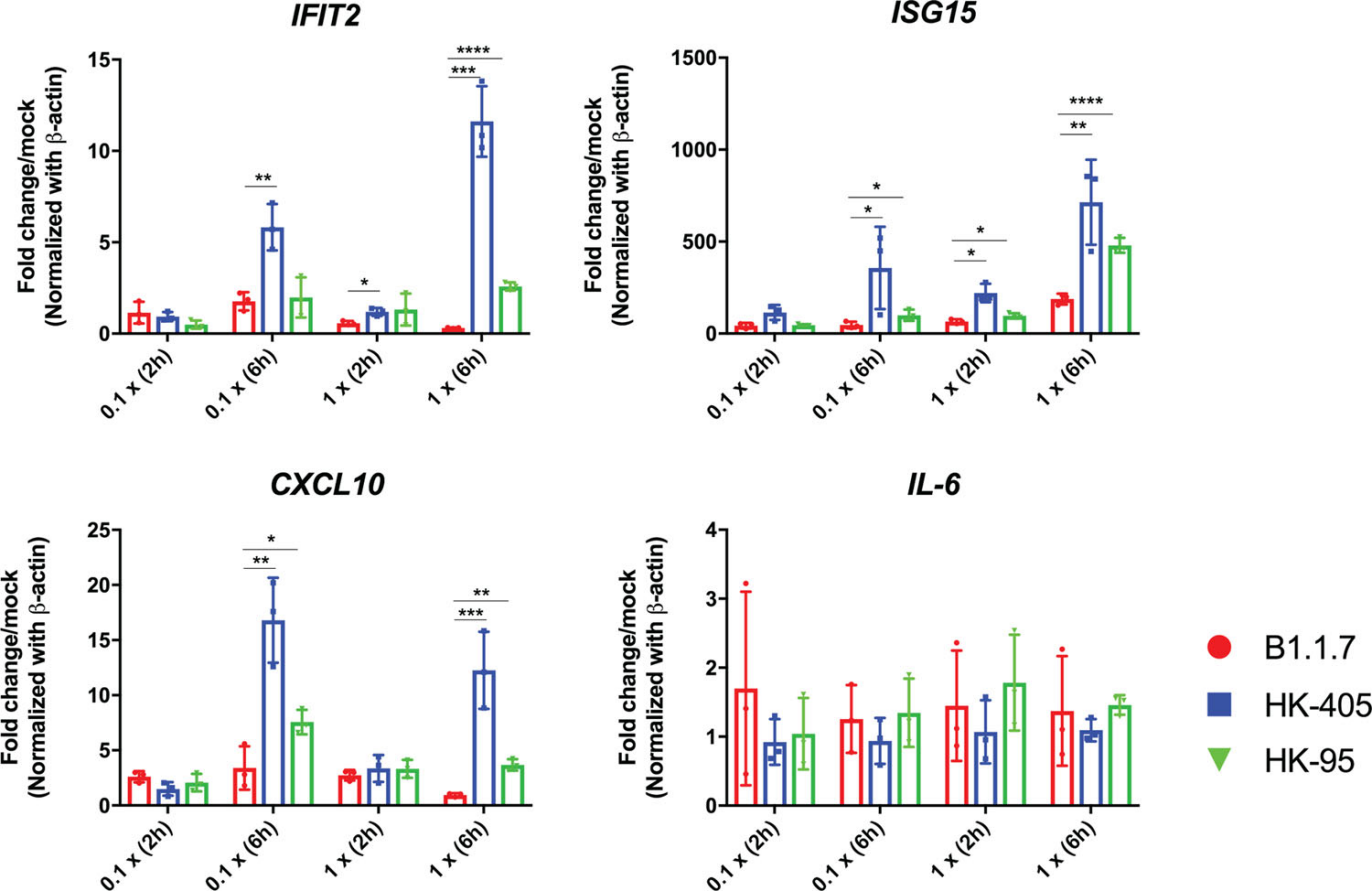
Calu-3 cells response to B.1.1.7 drive less macrophage activation. M0 THP1 cells were treated with filtered, virus-free conditioned media supernatants (prepared from Calu-3 cells infected with different variants as indicated) at dilution 0.1x (1:10 dilution with DMEM) and 1x (no dilution) for 2 or 6 h. Expression of immune genes were then measured by qPCR analysis. Data shown are mean ±SD, *n* = 3, statistical comparison are made by Student’s *t* test.

Although we observed viral load difference in hamster nasal cavities on day 1 postinfection with low dose inoculum, almost no replication difference of the variants was measured in all tested cell cultures. We speculated that this discrepancy might be explained by the innate immune microenvironment in which crosstalk between the epithelial and immune cells occurs. Variants acquire different abilities to antagonise the host innate immune system, which could directly affect its own dissemination. During the initial stage of infection, the infected epithelial cells along the nasal cavity could express stimulatory factors to recruit and trigger immune cells to release inflammatory signals to prime neighbouring uninfected cells response to infection. To imitate this situation, we treated THP-1 cells with filtered, virus-free conditioned media supernatants from different SARS-CoV-2 infected Calu-3 cells. It has been proposed that increased expression of the key viral innate antagonists, like Orf9b and Orf6, might contribute to immune evasion of the Alpha variant^20^. Consistently, we demonstrated that the immune stimulatory activity of conditioned media in the Alpha variant infected Calu-3 cells was much attenuated than that of the earlier D614G variants (Fig. 5), suggesting that the viral mutations in the Alpha variant might acquire enhanced immune antagonism to support a higher replication rate in in vivo systems.

SARS-CoV-2 VOCs have been emerging in many countries in the past few months, and it is crucial to establish relevant experimental models to characterise existing and new variants in terms of transmissibility, disease severity and vaccine efficacy, and to evaluate therapeutic interventions. In this report, by using a lower infectious dose, we demonstrate that the Alpha variant exhibits higher infectivity and/or replication efficiency in the nasal epithelium. Our data, albeit based on only one of the VOCs, show that novel VOCs can transmit more efficiently than other pre-existing strains with fewer infectious viral particles. Further work to compare transmission ability with other emerging VOCs and investigation of routes of transmission are required. A better understanding of SARS-CoV-2 transmission dynamics is important for designing appropriate strategies for a more effective response to emerging variants of SARS-CoV-2.

## Methods

### Viruses

The SARS-CoV-2 isolates HK-95 (MT835143), HK-405 (MW856793), B.1.1.7 (MW856794) and HK-15 (MT835141) were isolated from specimens obtained from four laboratory-confirmed COVID-19 patients using Vero E6 cells (ATCC; CRL-15786). All experiments involving SARS-CoV-2 viruses were conducted in a Biosafety Level-3 laboratory. For animal challenge, viral stocks were prepared after two serial passages of isolated virus in Vero E6 cells in Dulbecco’s Modified Eagle Medium (DMEM) (Thermo Fisher Scientific) supplemented with 5% foetal bovine serum (Thermo Fisher Scientific), and 100 IU penicillin G/ml and 100 ml streptomycin sulphate/ml (Thermo Fisher Scientific), and were divided into aliquots. The aliquots were then sequenced, and viral titres were determined by plaque assay using Vero E6 cells. All viruses used in this study were from the same batch of aliquots and used without freeze-thawing. Viral RNAs were obtained from the supernatants of infected cells and then isolated using the QIAamp RNA Viral kit (Qiagen) and subjected to whole viral genome sequencing.

### Whole viral genome sequencing

Viral RNA was extracted from the supernatant of the infected Vero E6 cells using the QIAamp RNA Viral kit (Qiagen). 140ul of the sample was treated with 560 µl of AVL, containing carrier RNA, and then mixed with 560 µl of absolute ethanol. The sample was then transferred into QiaAmp spin column, centrifuged, washed with wash buffer provided and then eluted with 20 ul of DEPC-treated water. Viral RNA was then treated using the TURBO DNA-free Kit (ThermoFisher Scientific) to remove residual host DNA, and then reverse-transcribed using SuperScript IV reverse transcriptase (Invitrogen). The viral cDNA was then enriched through multiplex tiling polymerase chain reaction (PCR), as described in the ARTIC network (https://artic.network/ncov-2019). The PCR contained a pool of 98 primer pairs, which generated overlapping 400-bp amplicons across the entire genome of SARS-CoV-2 (accession no: NC_045512) (primer sequences are shown in Supplementary Table 1). The final PCR mastermix (25 µL) included 2.5 µL of cDNA, 5.0 µL of 5X Q5 Reaction Buffer, 0.5 µL of 10 mM dNTP mix, 0.25 µL of Q5 Hot Start DNA Polymerase (New England Biolabs) and 3.6 µL of 10 µM primer pool 1 or 2, plus 13.15 µL of nuclease-free water. The mixtures were incubated at 98 °C for 30 s, followed by 35 cycles at 98 °C for 15 s and 65 °C for 5 min. The PCR amplicons were then purified using 1X Agencourt AMPure XP beads (Beckman Coulter).

A total of 100 ng of multiplex PCR amplicons were subjected to library preparation and dual-indexing using a KAPA HyperPrep Kit and a Unique Dual-Indexed Adapter Kit (Roche Applied Science) according to the manufacturer’s instructions. Ligated libraries were then enriched by six-cycle PCR amplification, followed by purification and size selection using AMPure XP beads (Beckman Coulter). The pooled libraries were sequenced using the MiSeq Reagent Kit V2 Nano on an Illumina MiSeq System. The Illumina MiSeq sequencing reads were then demultiplexed and mapped to the reference genome (accession number: NC_0.45512.2) using Samtools v1.7. Variants were called with Freebayes v1.0.0 (https://arxiv.org/abs/1207.3907) with the haploid setting, with a minimum base quality and depth of coverage of Q30 and 50x, respectively.

### Hamster infection

Female golden Syrian hamsters, aged 4–6 or 6–8 weeks old, were obtained from the LASEC, Chinese University of Hong Kong via the Centre for Comparative Medicine Research at the University of Hong Kong (HKU). All experiments were performed in a Biosafety Level-3 animal facility at the LKS Faculty of Medicine, HKU. Hamsters were housed in ventilated isolator cages (IsoCage N, Tecniplast) at a temperature of 21 °C, the humidity of 70%, and 12:12 dark/light cycles, with access to food and water ad libitum. Housing conditions and experimental procedures were approved by the Committee on the Use of Live Animals in Teaching and Research, HKU. Hamsters were anaesthetised with ketamine (150 mg/kg) and xylazine (10 mg/mg) via intraperitoneal injection and then intranasally inoculated with 50 µl of diluted virus stock. All hamsters were euthanized by intraperitoneal injection of pentobarbital at 200 mg/kg. Body weights were monitored daily for 4 days. Nasal washes were collected from hamsters daily for 4 consecutive days. Total nucleic acid was extracted from 70 ul of sample fluid using the QIAamp RNA Viral kit (Qiagen). In brief, sample was treated with 280 µl of AVL, containing carrier RNA, followed by 280 µl of 100% ethanol. The sample was then transferred into QiaAmp spin column, centrifuged, washed with buffer provided and then eluted with 10 µl of DEPC-treated water. 7.4 µl RNA was used for reverse transcription using MultiScribe Reverse Transcriptase (Thermofisher). cDNA was subsequently used for real-time qPCR using TB Green Premix Ex Taq (Tli RNase H Plus) (Takara). For the in vivo co-infection experiment, viral RNA from nasal washes was collected for sanger sequencing. All Hamsters were euthanized at 4 dpi, lung right lobes (superior, middle, and inferior) or nasal turbinates were homogenised in 1 ml of PBS. After centrifugation at 12,000 rpm for 10 min, the supernatant was harvested, and viral titres were determined by plaque assay using Vero E6 cells. Lung left superior lobes were fixed in 4% paraformaldehyde and then processed for paraffin embedding. The 4 µm tissue sections were stained with haematoxylin and eosin for histopathological examination. Immunohistology was performed using mouse anti-coronavirus nucleocapsid antibody (1:200, 40143-MM05, Sinobiological). Images were obtained with an Olympus BX53 semimotorised fluorescence microscope using cellSens imaging 342 software.

### Transmission experiment

Three 4–6-week-old hamsters were inoculated intranasally with 10 PFU (50 μl) of virus. At 24 h after infection, each infected hamster was placed in a specially designed cage inside a ventilated cage. Two to three naive hamsters were placed on the other side of the cage with 5 cm separation by a double-layered divider to allow free airflow (70 air changes per hour). After two hours of exposure, each naïve hamsters (*n* = 8 per group) was placed in a new individual cage. To assess viral replication in nasal turbinates, we determined the virus load in the nasal wash specimens collected from exposed hamsters on day 2 and day 4 after transmission. Viral RNA was extracted from 70 μl of each nasal wash sample.

### In-vitro competitive fitness assay

Calu-3 cells in Dulbecco’s Modified Eagle Medium (DMEM) (Thermo Fisher Scientific) supplemented with 5% foetal bovine serum (Thermo Fisher Scientific), and 100 IU penicillin G/ml and 100 ml streptomycin sulphate/ml (Thermo Fisher Scientific) were infected with MOI of 0.1 of B.1.1.7 and another variant of the D614G lineage, either HK-95 or HK-405 mixture at 1:1 ratio. Following 1 h incubation, the cultures were washed thrice with PBS and cultured for 3 days. To passage the progeny viruses, the virus samples were continuously passaged three times in Calu-3 cells. Viral RNAs were obtained from the supernatants of infected cells and then isolated using the QIAamp RNA Viral kit (Qiagen). A 901 bp fragment containing the N501Y site was amplified from each RNA sample by RT-PCR using primer set: 5’- GAAGTCAGACAAATCGCTCCAG-3’ and 5’-GCAACTGAATTTTCTGCACCA-3’. The amplicon was purified by NucleoSpin® Gel and PCR Clean-Up (Takara) for Sanger sequencing.

### Treatment of THP-1 cells with virus-free conditioned medium

The human monocytic cell line THP-1 was maintained in RPMI medium supplemented with 10% FBS, NEAA, L-glutamate, sodium pyruvate and penicillin/streptomycin. To differentiate into M0 macrophage-like cells, THP-1 cells were cultured at 100,000 cells per well in a 96-well plate in the presence of 50 ng/ml phorbol 12-myristate 13-acetate (PMA) for 2 days, followed by washing out in PMA-free medium for 2 days. To generate virus-free conditioned media, Calu-3 cells were mock-infected or infected with Sars-Cov-2 variants at 0.01 MOI, and supernatant were harvested at 48 h postinfection, filtered using Amicon Ultra 100 K (Millipore). Then, M0 THP-1 cells were exposed to the conditioned medium. RNA from treated THP-1 cells were extracted using RNAzol (Takara). Expression of immune genes were then measured by qRT-PCR analysis. Data shown are mean and standard deviation of 3 replicates per condition. *P*-values were determined by Student’s t-test.

### RT-qPCR

RNA was extracted using RNAzol (Takara) and cDNA was synthesised using PrimeScript RT Reagent Kit with gDNA Eraser (Takara). RT–qPCR was performed using TB Green Premix Ex Taq (Takara). The PCR program consisted of a pre-incubation for 10 min at 95 °C, followed by 40 cycles of denaturation for 10 sec at 95 °C, annealing for 10 sec at 60 °C and extension for 10 sec at 72 °C. Host gene expression was determined using the 2-ΔΔCt method and normalised to beta-actin expression. RT-qPCR primers used in this study are listed in Table 1.

**Table 1:** Primers used for RT-qPCR.

Target	Primer sequence
human_beta-actin	Fwd: 5’-GGAAATCGTGCGTGACATTA-3'
	Rev: 5’-AGGAGGAAGGCTGGAAGAG-3'
human_IFIT2	Fwd: 5’-AGGCTTTGCATGTCTTGG-3'
	Rev: 5’-GAGTCTTCATCTGCTTGTTGC-3'
human_Cxcl10	Fwd: 5’-GTGGCATTCAAGGAGTACCTC-3'
	Rev: 5’-TGATGGCCTTCGATTCTGGATT-3'
human_IL6	Fwd: 5’-CCAGGAGAAGATTCCAAAGATG-3'
	Rev: 5’-GGAAGGTTCAGGTTGTTTTCTG-3'
human_ISG15	Fwd: 5’-CGCAGATCACCCAGAAGATCG-3'
	Rev: 5’-TTCGTCGCATTTGTCCACCA-3'
nCov-spike	Fwd: 5’-GAAGTCAGACAAATCGCTCCAG-3'
	Rev: 5’-GCAACTGAATTTTCTGCACCA-3'

### Statistics and reproducibility

Graphpad prism Version 7.05. (GraphPad) was used for all statistical analysis. The number of animals and independent experiments that were performed is indicated in the legends to figures. Statistical significance was determined using Student’s t-test. *P* values of ≤0.05 were considered as statistically significant.

### Reporting Summary

Further information on research design is available in the Nature Research Reporting Summary linked to this article.

## Supplementary information

Supplementary Information

Description of Additional Supplementary Files

Supplementary Data 1

Reporting Summary

## Data Availability

Source data for all figures are provided in Supplementary Data file 1. Sequencing data of all the viral strains used in the current study were deposited in Genbank with the following accession number: HK-95 (MT835143), HK-405 (MW856793), B.1.1.7 (MW856794) and HK-15 (MT835141).

## Code availability

No in-house computer code or script was used in this study. The programs used for analysis of sequencing data are open-source and published, e.g. Samtools v1.7 and Freebayes v1.0.0.
