# Supplementary material for: Low dose inocula of SARS-CoV-2 Alpha variant transmits more efficiently than earlier variants in hamsters
Source: Commun Biol. 2021 Sep 20;4:1102. doi: 10.1038/s42003-021-02640-x (PMC8452646; doi:10.1038/s42003-021-02640-x)
Supplement: Supplementary file 1 — Supplementary Information [file 42003_2021_2640_MOESM1_ESM.pdf]

Supplementary Figure 1. Viral replication in nasal turbinates and lungs of hamsters infected with different dose inocula of B.1.1.7. Hamsters (2 per group) were each inoculated intranasally with different dose inoculums of B.1.1.7 as indicated. All hamsters were euthanized on one day post-infection for viral titration. Horizontal lines indicate the overall mean of average viral titre values per group. Statistical significance was calculated by Student's t-test; * denotes $p<0.05$.

Supplementary Figure 2. (a) Set-up of the hamster transmission study. Donor hamsters were given 10PFU of each virus, HK-405 or B.1.1.7. After 24 hours post infection, two to three naive hamsters were exposed to one inoculated donor hamster for 2 hours. The infected hamster and the exposed hamsters were separated by a 5-cm space to avoid direct contact. Transmission was done by holding the hamsters inside individually ventilated cages (IsoCage N, Techniplast) with 70 air changes per hour. After exposure, each hamster was single housed in an individual cage. (b) Validation of the inoculation dose by plaque assay. (c) Image of hamsters in the cage during transmission study in the animal BSL-3 facility.

Supplementary Figure 1

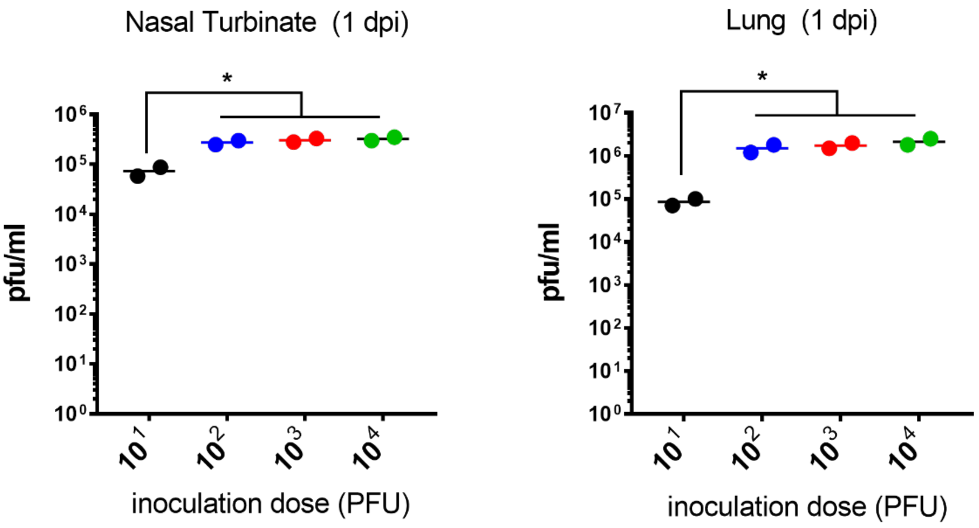

Supplementary Figure 2

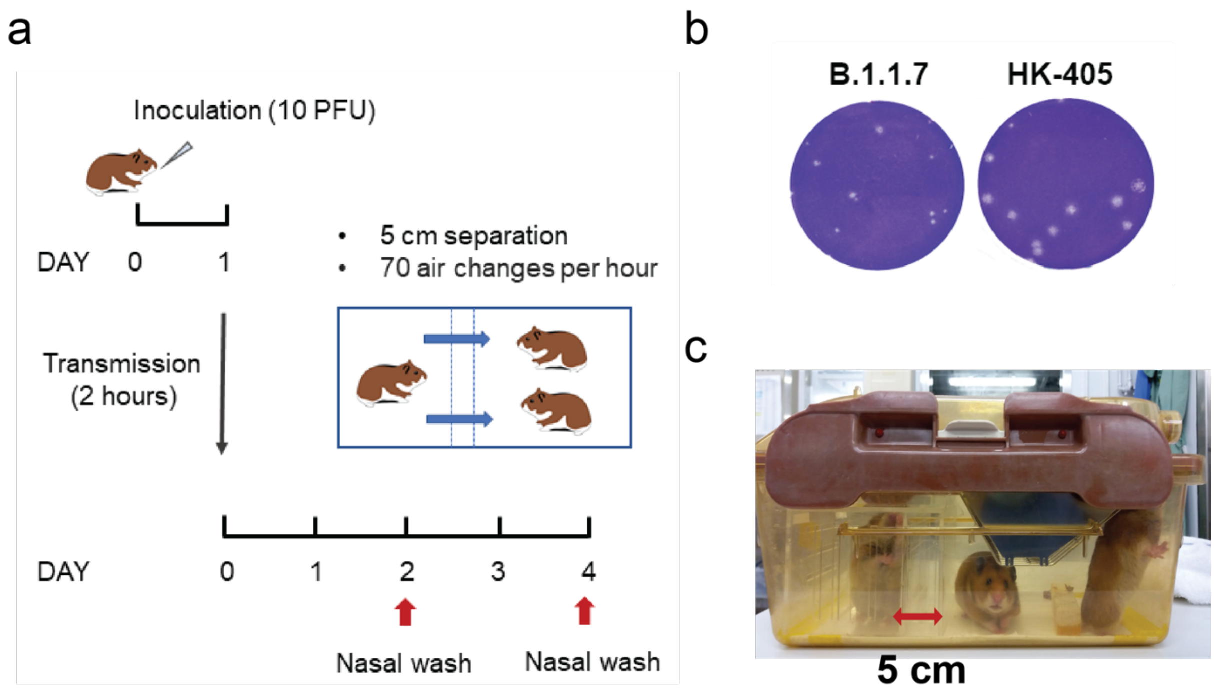

Supplementary Table 1: Primers used in multiplex PCRs for SARS-CoV-2 genome amplification

Primer name	Sequence	Primer Start[a]	Primer end[a]	PCR Pool	Primer length	GC contents	Tm
nCoV-2019_1_LEFT	ACCAACCAACTTTCGATCTCTTGT	30	54	Pool 1	24	41.67	60.69
nCoV-2019_1_RIGHT	CATCTTTAAGATGTTGACGTGCCTC	385	410	Pool 1	25	44	60.45
nCoV-2019_2_LEFT	CTGTTTTACAGGTTCGCGACGT	320	342	Pool 2	22	50	61.67
nCoV-2019_2_RIGHT	TAAGGATCAGTGCCAAGCTCGT	704	726	Pool 2	22	50	61.74
nCoV-2019_3_LEFT	CGGTAATAAAGGAGCTGGTGGC	642	664	Pool 1	22	54.55	61.32
nCoV-2019_3_RIGHT	AAGGTGTCTGCAATTCATAGCTCT	1004	1028	Pool 1	24	41.67	60.32
nCoV-2019_4_LEFT	GGTGTATACTGCTGCCGTGAAC	943	965	Pool 2	22	54.55	61.56
nCoV-2019_4_RIGHT	CACAAGTAGTGGCACCTTCTTTAGT	1312	1337	Pool 2	25	44	60.97
nCoV-2019_5_LEFT	TGGTGAAACTTCATGGCAGACG	1242	1264	Pool 1	22	50	61.39
nCoV-2019_5_RIGHT	ATTGATGTTGACTTTCTCTTTTTGGAGT	1623	1651	Pool 1	28	32.14	60.17
nCoV-2019_6_LEFT	GGTGTTGTTGGAGAAGGTTCCG	1573	1595	Pool 2	22	54.55	61.64
nCoV-2019_6_RIGHT	TAGCGGCCTTCTGTAAAACACG	1942	1964	Pool 2	22	50	61.18
nCoV-2019_7_LEFT	ATCAGAGGCTGCTCGTGTTGTA	1875	1897	Pool 1	22	50	61.73
nCoV-2019_7_RIGHT	TGCACAGGTGACAATTTGTCCA	2247	2269	Pool 1	22	45.45	60.95
nCoV-2019_8_LEFT	AGAGTTTCTTAGAGACGGTTGGGA	2181	2205	Pool 2	24	45.83	61
nCoV-2019_8_RIGHT	GCTTCAACAGCTTCACTAGTAGGT	2568	2592	Pool 2	24	45.83	60.56
nCoV-2019_9_LEFT	TCCCACAGAAGTGTTAACAGAGGA	2505	2529	Pool 1	24	45.83	61.18
nCoV-2019_9_RIGHT	ATGACAGCATCTGCCACAACAC	2882	2904	Pool 1	22	50	61.71
nCoV-2019_10_LEFT	TGAGAAGTGCTCTGCCTATACAGT	2826	2850	Pool 2	24	45.83	61.12
nCoV-2019_10_RIGHT	TCATCTAACCAATCTTCTTCTTGCTCT	3183	3210	Pool 2	27	37.04	60.31
nCoV-2019_11_LEFT	GGAATTTGGTGCCACTTCTGCT	3144	3166	Pool 1	22	50	61.66
nCoV-2019_11_RIGHT	TCATCAGATTCAACTTGCATGGCA	3507	3531	Pool 1	24	41.67	61.35
nCoV-2019_12_LEFT	AAACATGGAGGAGGTGTTGCAG	3460	3482	Pool 2	22	50	61.08
nCoV-2019_12_RIGHT	TTCACTCTTCATTTCCAAAAAGCTTGA	3826	3853	Pool 2	27	33.33	60.36
nCoV-2019_13_LEFT	TCGCACAAATGTCTACTTAGCTGT	3771	3795	Pool 1	24	41.67	60.56
nCoV-2019_13_RIGHT	ACCACAGCAGTTAAAACACCCT	4142	4164	Pool 1	22	45.45	60.36
nCoV-2019_14_LEFT	CATCCAGATTCTGCCACTCTTGT	4054	4077	Pool 2	23	47.83	60.62
nCoV-2019_14_RIGHT	AGTTTCCACACAGACAGGCATT	4428	4450	Pool 2	22	45.45	60.42
nCoV-2019_15_LEFT	ACAGTGCTTAAAAAGTGTAAAAGTGCC	4294	4321	Pool 1	27	37.04	61.32
nCoV-2019_15_RIGHT	AACAGAAACTGTAGCTGGCACT	4674	4696	Pool 1	22	45.45	60.16
nCoV-2019_16_LEFT	AATTTGGAAGAAGCTGCTCGGT	4636	4658	Pool 2	22	45.45	60.82
nCoV-2019_16_RIGHT	CACAACTTGCGTGTGGAGGTTA	4995	5017	Pool 2	22	50	61.32
nCoV-2019_17_LEFT	CTTCTTTCTTTGAGAGAAGTGAGGACT	4939	4966	Pool 1	27	40.74	60.69
nCoV-2019_17_RIGHT	TTTGTTGGAGTGTTAACAATGCAGT	5296	5321	Pool 1	25	36	60.11
nCoV-2019_18_LEFT	TGGAAATACCCACAAGTTAATGGTTTAAC	5230	5259	Pool 2	29	34.48	60.69
nCoV-2019_18_RIGHT	AGCTTGTTTACCACACGTACAAGG	5620	5644	Pool 2	24	45.83	61.51
nCoV-2019_19_LEFT	GCTGTTATGTACATGGGCACACT	5563	5586	Pool 1	23	47.83	61.18
nCoV-2019_19_RIGHT	TGTCCAACTTAGGGTCAATTTCTGT	5932	5957	Pool 1	25	40	60.4
nCoV-2019_20_LEFT	ACAAAGAAAACAGTTACACAACAACCA	5867	5894	Pool 2	27	33.33	60.68
nCoV-2019_20_RIGHT	ACGTGGCTTTATTAGTTGCATTGTT	6247	6272	Pool 2	25	36	60.28
nCoV-2019_21_LEFT	TGGCTATTGATTATAAACACTACACACCC	6167	6196	Pool 1	29	37.93	61.49
nCoV-2019_21_RIGHT	TAGATCTGTGTGGCCAACCTCT	6528	6550	Pool 1	22	50	60.83
nCoV-2019_22_LEFT	ACTACCGAAGTTGTAGGAGACATTATACT	6466	6495	Pool 2	29	37.93	61.25
nCoV-2019_22_RIGHT	ACAGTATTCTTTGCTATAGTAGTCGGC	6846	6873	Pool 2	27	40.74	60.73
nCoV-2019_23_LEFT	ACAACTACTAACATAGTTACACGGTGT	6718	6745	Pool 1	27	37.04	60.26
nCoV-2019_23_RIGHT	ACCAGTACAGTAGGTTGCAATAGTG	7092	7117	Pool 1	25	44	60.57
nCoV-2019_24_LEFT	AGGCATGCCTTCTTACTGTACTG	7035	7058	Pool 2	23	47.83	60.37
nCoV-2019_24_RIGHT	ACATTCTAACCATAGCTGAAATCGGG	7389	7415	Pool 2	26	42.31	61.19
nCoV-2019_25_LEFT	GCAATTGTTTTTCAGCTATTTTGCAGT	7305	7332	Pool 1	27	33.33	60.73

nCoV-2019_25_RIGHT	ACTGTAGTGACAAGTCTCTCGCA	7671	7694	Pool 1	23	47.83	61.3
nCoV-2019_26_LEFT	TTGTGATACATTCTGTGCTGGTAGT	7626	7651	Pool 2	25	40	60.28
nCoV-2019_26_RIGHT	TCCGCACTATCACCAACATCAG	7997	8019	Pool 2	22	50	60.42
nCoV-2019_27_LEFT	ACTACAGTCAGCTTATGTGTCAACC	7943	7968	Pool 1	25	44	60.8
nCoV-2019_27_RIGHT	AATACAAGCACCAAGGTCACGG	8319	8341	Pool 1	22	50	61.13
nCoV-2019_28_LEFT	ACATAGAAGTTACTGGCGATAGTTGT	8249	8275	Pool 2	26	38.46	60.13
nCoV-2019_28_RIGHT	TGTTTAGACATGACATGAACAGGTGT	8635	8661	Pool 2	26	38.46	60.91
nCoV-2019_29_LEFT	ACTTGTGTTCCTTTTTGTTGCTGC	8595	8619	Pool 1	24	41.67	61.39
nCoV-2019_29_RIGHT	AGTGTACTCTATAAGTTTTGATGGTGTGT	8954	8983	Pool 1	29	34.48	60.69
nCoV-2019_30_LEFT	GCACAACTAATGGTGACTTTTTGCA	8888	8913	Pool 2	25	40	61.19
nCoV-2019_30_RIGHT	ACCACTAGTAGATACACAAACACCAG	9245	9271	Pool 2	26	42.31	60.3
nCoV-2019_31_LEFT	TTCTGAGTACTGTAGGCACGGC	9204	9226	Pool 1	22	54.55	62.03
nCoV-2019_31_RIGHT	ACAGAATAAACACCAGGTAAGAATGAGT	9557	9585	Pool 1	28	35.71	60.69
nCoV-2019_32_LEFT	TGGTGAATACAGTCATGTAGTTGCC	9477	9502	Pool 2	25	44	61.09
nCoV-2019_32_RIGHT	AGCACATCACTACGCAACTTTAGA	9834	9858	Pool 2	24	41.67	60.56
nCoV-2019_33_LEFT	ACTTTTGAAGAAGCTGCGCTGT	9784	9806	Pool 1	22	45.45	61.58
nCoV-2019_33_RIGHT	TGGACAGTAAACTACGTCATCAAGC	10146	10171	Pool 1	25	44	61.08
nCoV-2019_34_LEFT	TCCCATCTGGTAAAGTTGAGGGT	10076	10099	Pool 2	23	47.83	61.02
nCoV-2019_34_RIGHT	AGTGAAATTGGGCCTCATAGCA	10437	10459	Pool 2	22	45.45	60.03
nCoV-2019_35_LEFT	TGTTCGCATTCAACCAGGACAG	10362	10384	Pool 1	22	50	61.39
nCoV-2019_35_RIGHT	ACTTCATAGCCACAAGGTTAAAGTCA	10737	10763	Pool 1	26	38.46	60.69
nCoV-2019_36_LEFT	TTAGCTTGGTTGTACGCTGCTG	10666	10688	Pool 2	22	50	61.44
nCoV-2019_36_RIGHT	GAACAAAGACCATTGAGTACTCTGGA	11048	11074	Pool 2	26	42.31	60.74
nCoV-2019_37_LEFT	ACACACCACTGGTTGTTACTCAC	10999	11022	Pool 1	23	47.83	60.93
nCoV-2019_37_RIGHT	GTCCACACTCTCCTAGCACCAT	11372	11394	Pool 1	22	54.55	61.48
nCoV-2019_38_LEFT	ACTGTGTTATGTATGCATCAGCTGT	11306	11331	Pool 2	25	40	60.86
nCoV-2019_38_RIGHT	CACCAAGAGTCAGTCTAAAGTAGCG	11668	11693	Pool 2	25	48	61.13
nCoV-2019_39_LEFT	AGTATTGCCCTATTTTCTTCATAACTGGT	11555	11584	Pool 1	29	34.48	61
nCoV-2019_39_RIGHT	TGTAACTGGACACATTGAGCCC	11927	11949	Pool 1	22	50	60.55
nCoV-2019_40_LEFT	TGCACATCAGTAGTCTTACTCTCAGT	11863	11889	Pool 2	26	42.31	61.25
nCoV-2019_40_RIGHT	CATGGCTGCATCACGGTCAAAT	12234	12256	Pool 2	22	50	62.09
nCoV-2019_41_LEFT	GTTCCCTTCCATCATATGCAGCT	12110	12133	Pool 1	23	47.83	60.75
nCoV-2019_41_RIGHT	TGGTATGACAACCATTAGTTTGGCT	12465	12490	Pool 1	25	40	60.75
nCoV-2019_42_LEFT	TGCAAGAGATGGTTGTGTTCCC	12417	12439	Pool 2	22	50	61.08
nCoV-2019_42_RIGHT	CCTACCTCCCTTTGTTGTGTTGT	12779	12802	Pool 2	23	47.83	60.69
nCoV-2019_43_LEFT	TACGACAGATGTCTTGTGCTGC	12710	12732	Pool 1	22	50	60.93
nCoV-2019_43_RIGHT	AGCAGCATCTACAGCAAAAGCA	13074	13096	Pool 1	22	45.45	61.14
nCoV-2019_44_LEFT	TGCCACAGTACGTCTACAAGCT	13005	13027	Pool 2	22	50	61.66
nCoV-2019_44_RIGHT	AACCTTTCCACATACCGCAGAC	13378	13400	Pool 2	22	50	60.87
nCoV-2019_45_LEFT	TACCTACAACTTGTGCTAATGACCC	13319	13344	Pool 1	25	44	60.57
nCoV-2019_45_RIGHT	AAATTGTTTCTTCATGTTGGTAGTTAGAGA	13669	13699	Pool 1	30	30	60.01
nCoV-2019_46_LEFT	TGTCGCTTCCAAGAAAAGGACG	13599	13621	Pool 2	22	50	61.38
nCoV-2019_46_RIGHT	CACGTTCACCTAAGTTGGCGTA	13962	13984	Pool 2	22	50	60.86
nCoV-2019_47_LEFT	AGGACTGGTATGATTTTGTAGAAAACCC	13918	13946	Pool 1	28	39.29	61.42
nCoV-2019_47_RIGHT	AATAACGGTCAAAGAGTTTTAACCTCTC	14271	14299	Pool 1	28	35.71	60.06
nCoV-2019_48_LEFT	TGTTGACACTGACTTAACAAAGCCT	14207	14232	Pool 2	25	40	61.09
nCoV-2019_48_RIGHT	TAGATTACCAGAAGCAGCGTGC	14579	14601	Pool 2	22	50	60.74
nCoV-2019_49_LEFT	AGGAATTACTTGTGTATGCTGCTGA	14545	14570	Pool 1	25	40	60.57

nCoV-2019_49_RIGHT	TGACGATGACTTGGTTAGCATTAATACA	14898	14926	Pool 1	28	35.71	61.05
nCoV-2019_50_LEFT	GTTGATAAGTACTTTGATTGTTACGATGGT	14865	14895	Pool 2	30	33.33	60.59
nCoV-2019_50_RIGHT	TAACATGTTGTGCCAACCACCA	15224	15246	Pool 2	22	45.45	60.95
nCoV-2019_51_LEFT	TCAATAGCCGCCACTAGAGGAG	15171	15193	Pool 1	22	54.55	61.34
nCoV-2019_51_RIGHT	AGTGCATTAACATTGGCCGTGA	15538	15560	Pool 1	22	45.45	61.14
nCoV-2019_52_LEFT	CATCAGGAGATGCCACAACTGC	15481	15503	Pool 2	22	54.55	61.83
nCoV-2019_52_RIGHT	GTTGAGAGCAAAATTCATGAGGTCC	15861	15886	Pool 2	25	44	60.62
nCoV-2019_53_LEFT	AGCAAAATGTTGGACTGAGACTGA	15827	15851	Pool 1	24	41.67	60.69
nCoV-2019_53_RIGHT	AGCCTCATAAAACTCAGGTTCCC	16186	16209	Pool 1	23	47.83	60.31
nCoV-2019_54_LEFT	TGAGTTAACAGGACACATGTTAGACA	16118	16144	Pool 2	26	38.46	60.18
nCoV-2019_54_RIGHT	AACCAAAAACTTGTCCATTAGCACA	16485	16510	Pool 2	25	36	60.11
nCoV-2019_55_LEFT	ACTCAACTTTACTTAGGAGGTATGAGCT	16416	16444	Pool 1	28	39.29	61.43
nCoV-2019_55_RIGHT	GGTGTACTCTCCTATTTGTACTTTACTGT	16804	16833	Pool 1	29	37.93	60.54
nCoV-2019_56_LEFT	ACCTAGACCACCACTTAACCGA	16748	16770	Pool 2	22	50	60.49
nCoV-2019_56_RIGHT	ACACTATGCGAGCAGAAGGGTA	17130	17152	Pool 2	22	50	61.21
nCoV-2019_57_LEFT	ATTCTACACTCCAGGGACCACC	17065	17087	Pool 1	22	54.55	61.16
nCoV-2019_57_RIGHT	GTAATTGAGCAGGGTCGCCAAT	17430	17452	Pool 1	22	50	61.26
nCoV-2019_58_LEFT	TGATTTGAGTGTTGTCAATGCCAGA	17381	17406	Pool 2	25	40	61.44
nCoV-2019_58_RIGHT	CTTTTCTCCAAGCAGGGTTACGT	17738	17761	Pool 2	23	47.83	61.06
nCoV-2019_59_LEFT	TCACGCATGATGTTTCATCTGCA	17674	17697	Pool 1	23	43.48	61.42
nCoV-2019_59_RIGHT	AAGAGTCCTGTTACATTTTCAGCTTG	18036	18062	Pool 1	26	38.46	60.02
nCoV-2019_60_LEFT	TGATAGAGACCTTTATGACAAGTTGCA	17966	17993	Pool 2	27	37.04	60.53
nCoV-2019_60_RIGHT	GGTACCAACAGCTTCTCTAGTAGC	18324	18348	Pool 2	24	50	60.44
nCoV-2019_61_LEFT	TGTTTATCACCCGCGAAGAAGC	18253	18275	Pool 1	22	50	61.5
nCoV-2019_61_RIGHT	ATCACATAGACAACAGGTGCGC	18650	18672	Pool 1	22	50	61.25
nCoV-2019_62_LEFT	GGCACATGGCTTTGAGTTGACA	18596	18618	Pool 2	22	50	61.91
nCoV-2019_62_RIGHT	GTTGAACCTTTCTACAAGCCGC	18957	18979	Pool 2	22	50	60.35
nCoV-2019_63_LEFT	TGTTAAGCGTGTTGACTGGACT	18896	18918	Pool 1	22	45.45	60.16
nCoV-2019_63_RIGHT	ACAAACTGCCACCATCACAACC	19275	19297	Pool 1	22	50	61.85
nCoV-2019_64_LEFT	TCGATAGATATCCTGCTAATTCCATTGT	19204	19232	Pool 2	28	35.71	60.11
nCoV-2019_64_RIGHT	AGTCTTGTAAAAGTGTTCCAGAGGT	19591	19616	Pool 2	25	40	60.1
nCoV-2019_65_LEFT	GCTGGCTTTAGCTTGTGGGTTT	19548	19570	Pool 1	22	50	61.92
nCoV-2019_65_RIGHT	TGTCAGTCATAGAACAAACACCAATAGT	19911	19939	Pool 1	28	35.71	60.9
nCoV-2019_66_LEFT	GGGTGTGGACATTGCTGCTAAT	19844	19866	Pool 2	22	50	61.21
nCoV-2019_66_RIGHT	TCAATTTCCATTTGACTCCTGGGT	20231	20255	Pool 2	24	41.67	60.45
nCoV-2019_67_LEFT	GTTGTCCAACAATTACCTGAAACTTACT	20172	20200	Pool 1	28	35.71	60.43
nCoV-2019_67_RIGHT	CAACCTTAGAAACTACAGATAAATCTTGGG	20542	20572	Pool 1	30	36.67	60.4
nCoV-2019_68_LEFT	ACAGGTTCATCTAAGTGTGTGTGT	20472	20496	Pool 2	24	41.67	60.14
nCoV-2019_68_RIGHT	CTCCTTTATCAGAACCAGCACCA	20867	20890	Pool 2	23	47.83	60.31
nCoV-2019_69_LEFT	TGTCGCAAAATATACTCAACTGTGTCA	20786	20813	Pool 1	27	37.04	61.43
nCoV-2019_69_RIGHT	TCTTTATAGCCACGGAACCTCCA	21146	21169	Pool 1	23	47.83	61.14
nCoV-2019_70_LEFT	ACAAAAGAAAATGACTCTAAAGAGGGTTT	21075	21104	Pool 2	29	31.03	60.13
nCoV-2019_70_RIGHT	TGACCTTCTTTTAAAGACATAACAGCAG	21427	21455	Pool 2	28	35.71	60.27
nCoV-2019_71_LEFT	ACAAATCCAATTCAGTTGTCTTCCTATTC	21357	31386	Pool 1	29	34.48	60.54

nCoV-2019_71_RIGHT	TGGAAAAGAAAGGTAAGAACAAGTCCT	21716	21743	Pool 1	27	37.04	60.8
nCoV-2019_72_LEFT	ACACGTGGTGTTTATTACCCTGAC	21658	21682	Pool 2	24	45.83	61.04
nCoV-2019_72_RIGHT	ACTCTGAACTCACTTTCCATCCAAC	22013	22038	Pool 2	25	44	60.97
nCoV-2019_73_LEFT	CAATTTTGTAATGATCCATTTTTGGGTGT	21961	21990	Pool 1	29	31.03	60.29
nCoV-2019_73_RIGHT	CACCAGCTGTCCAACCTGAAGA	22324	22346	Pool 1	22	54.55	62.45
nCoV-2019_74_LEFT	ACATCACTAGGTTTCAAACTTTACTTGC	22262	22290	Pool 2	28	35.71	60.68
nCoV-2019_74_RIGHT	GCAACACAGTTGCTGATTCTCTTC	22626	22650	Pool 2	24	45.83	60.85
nCoV-2019_75_LEFT	AGAGTCCAACCAACAGAATCTATTGT	22516	22542	Pool 1	26	38.46	60.24
nCoV-2019_75_RIGHT	ACCACCAACCTTAGAATCAAGATTGT	22877	22903	Pool 1	26	38.46	60.69
nCoV-2019_76_LEFT	AGGGCAAACTGGAAAGATTGCT	22797	22819	Pool 2	22	45.45	60.76
nCoV-2019_76_RIGHT	ACACCTGTGCCTGTTAAACCAT	23192	23214	Pool 2	22	45.45	60.42
nCoV-2019_77_LEFT	CCAGCAACTGTTTGTGGACCTA	23122	23144	Pool 1	22	50	60.75
nCoV-2019_77_RIGHT	CAGCCCCTATTAAACAGCCTGC	23500	23522	Pool 1	22	54.55	61.59
nCoV-2019_78_LEFT	CAACTTACTCCTACTTGGCGTGT	23443	23466	Pool 2	23	47.83	60.55
nCoV-2019_78_RIGHT	TGTGTACAAAAACTGCCATATTGCA	23822	23847	Pool 2	25	36	60.22
nCoV-2019_79_LEFT	GTGGTGATTCAACTGAATGCAGC	23789	23812	Pool 1	23	47.83	60.92
nCoV-2019_79_RIGHT	CATTTCATCTGTGAGCAAAGGTGG	24145	24169	Pool 1	24	45.83	60.62
nCoV-2019_80_LEFT	TTGCCTTGGTGATATTGCTGCT	24078	24100	Pool 2	22	45.45	60.89
nCoV-2019_80_RIGHT	TGGAGCTAAGTTGTTTAACAAGCG	24443	24467	Pool 2	24	41.67	60.02
nCoV-2019_81_LEFT	GCACTTGGAAAACTTCAAGATGTGG	24391	24416	Pool 1	25	44	61.24
nCoV-2019_81_RIGHT	GTGAAGTTCTTTTCTTGTGCAGGG	24765	24789	Pool 1	24	45.83	60.73
nCoV-2019_82_LEFT	GGGCTATCATCTTATGTCCTTCCCT	24696	24721	Pool 2	25	48	61.52
nCoV-2019_82_RIGHT	TGCCAGAGATGTCACCTAAATCAA	25052	25076	Pool 2	24	41.67	60.02
nCoV-2019_83_LEFT	TCCTTTGCAACCTGAATTAGACTCA	24978	25003	Pool 1	25	40	60.46
nCoV-2019_83_RIGHT	TTTGACTCCTTTGAGCACTGGC	25347	25369	Pool 1	22	50	61.33
nCoV-2019_84_LEFT	TGCTGTAGTTGTCTCAAGGGCT	25279	25301	Pool 2	22	50	61.61
nCoV-2019_84_RIGHT	AGGTGTGAGTAAACTGTTACAAACAAC	25646	25673	Pool 2	27	37.04	60.36
nCoV-2019_85_LEFT	ACTAGCACTCTCCAAGGGTGTT	25601	25623	Pool 1	22	50	61.03
nCoV-2019_85_RIGHT	ACACAGTCTTTTACTCCAGATTCCC	25969	25994	Pool 1	25	44	60.51
nCoV-2019_86_LEFT	TCAGGTGATGGCACAACAAGTC	25902	25924	Pool 2	22	50	61.07
nCoV-2019_86_RIGHT	ACGAAAGCAAGAAAAAGAAGTACGC	26290	26315	Pool 2	25	40	61.01
nCoV-2019_87_LEFT	CGACTACTAGCGTGCCTTTGTA	26197	26219	Pool 1	22	50	60.16
nCoV-2019_87_RIGHT	ACTAGGTTCCATTGTTCAAGGAGC	26566	26590	Pool 1	24	45.83	60.81
nCoV-2019_88_LEFT	CCATGGCAGATTCCAACGGTAC	26520	26542	Pool 2	22	54.55	61.58
nCoV-2019_88_RIGHT	TGGTCAGAATAGTGCCATGGAGT	26890	26913	Pool 2	23	47.83	61.4
nCoV-2019_89_LEFT	GTACGCGTTCCATGTGGTCATT	26835	26857	Pool 1	22	50	61.5
nCoV-2019_89_RIGHT	ACCTGAAAGTCAACGAGATGAAACA	27202	27227	Pool 1	25	40	60.91
nCoV-2019_90_LEFT	ACACAGACCATTCCAGTAGCAGT	27141	27164	Pool 2	23	47.83	61.58
nCoV-2019_90_RIGHT	TGAAATGGTGAATTGCCCTCGT	27511	27533	Pool 2	22	45.45	60.82
nCoV-2019_91_LEFT	TCACTACCAAGAGTGTGTTAGAGGT	27446	27471	Pool 1	25	44	60.93
nCoV-2019_91_RIGHT	TTCAAGTGAGAACCAAAAGATAATAAGCA	27825	27854	Pool 1	29	31.03	60.03
nCoV-2019_92_LEFT	TTTGTGCTTTTTAGCCTTTCTGCT	27784	27808	Pool 2	24	37.5	60.14
nCoV-2019_92_RIGHT	AGGTTCCTGGCAATTAATTGTAAAAGG	28145	28172	Pool 2	27	37.04	60.53
nCoV-2019_93_LEFT	TGAGGCTGGTTCTAAATCACCCA	28081	28104	Pool 1	23	47.83	61.59
nCoV-2019_93_RIGHT	AGGTCTTCCTTGCCATGTTGAG	28442	28464	Pool 1	22	50	60.55
nCoV-2019_94_LEFT	GGCCCCAAGGTTTACCCAATAA	28394	28416	Pool 2	22	50	60.56
nCoV-2019_94_RIGHT	TTTGGCAATGTTGTTCCTTGAGG	28756	28779	Pool 2	23	43.48	60.18
nCoV-2019_95_LEFT	TGAGGGAGCCTTGAATACACCA	28677	28699	Pool 1	22	50	61.1

nCoV-2019_95_RIGHT	CAGTACGTTTTTGCCGAGGCTT	29041	29063	Pool 1	22	50	61.95
nCoV-2019_96_LEFT	GCCAACAACAACAAGGCCAAAC	28985	29007	Pool 2	22	50	61.82
nCoV-2019_96_RIGHT	TAGGCTCTGTTGGTGGGAATGT	29356	29378	Pool 2	22	50	61.36
nCoV-2019_97_LEFT	TGGATGACAAAGATCCAAATTTCAAAGA	29288	29316	Pool 1	28	32.14	60.22
nCoV-2019_97_RIGHT	ACACACTGATTAAAGATTGCTATGTGAG	29665	29693	Pool 1	28	35.71	60.17
nCoV-2019_98_LEFT	AACAATTGCAACAATCCATGAGCA	29486	29510	Pool 2	24	37.5	60.5
nCoV-2019_98_RIGHT	TTCTCCTAAGAAGCTATTAAAATCACATGG	29836	29866	Pool 2	30	33.33	60.01

[a] The nucleotide position is based on accession no. NC_045512.2
