# Supplementary material for: Low dose inocula of SARS-CoV-2 Alpha variant transmits more efficiently than earlier variants in hamsters
Source: Commun Biol. 2021 Sep 20;4:1102. doi: 10.1038/s42003-021-02640-x (PMC8452646; doi:10.1038/s42003-021-02640-x)
Supplement: Supplementary file 2 — Description of Additional Supplementary Files [file 42003_2021_2640_MOESM2_ESM.pdf]

Description of Additional Supplementary Files

File name: Supplementary Data 1.
Description: Source data underlying figures.
